# Effect of Gene-Based Warfarin Dosing on Anticoagulation Control and Clinical Events in a Real-World Setting

**DOI:** 10.3389/fphar.2019.01527

**Published:** 2020-01-17

**Authors:** Jinhua Zhang, Tingting Wu, Wenjun Chen, Jinglan Fu, Xiaotong Xia, Liangwan Chen

**Affiliations:** ^1^Department of Pharmacy, Fujian Medical University Union Hospital, Fuzhou, China; ^2^College of Pharmacy, Fujian Medical University, Fuzhou, China; ^3^Department of Cardiovascular Surgery, Fujian Medical University Union Hospital, Fuzhou, China

**Keywords:** pharmacogenetics, CYP2C9, precision medicine, warfarin, VKORC1

## Abstract

The *cytochrome P450 2C9* and *vitamin K epoxide reductase complex subunit 1* genotypes are associated with anticoagulation control and the clinical events in warfarin therapy. However, the clinical utility of gene-based warfarin dosing (GBWD) is controversial. We compared the anticoagulation control and clinical events related to warfarin with GBWD to those with clinically fixed dosing (CFD). A retrospective cohort study was conducted in a real-world setting. Of the 915 patients who were reviewed, 844 patients met the study-entry criteria; 413 cases were guided by GBWD using the International Warfarin Pharmacogenetic Consortium algorithm; 431 cases were guided by CFD (2.5 mg/day). The primary outcomes were the time needed to achieve the therapeutic International Normalized Ratio (INR) and the time in the therapeutic range (TTR) during a 3-month timeframe. The time needed to achieve the therapeutic INR (in days) for patients in the GBWD group was shorter than that for patients in the CFD group (10.21 ± 4.68 *vs.* 14.31 ± 8.26, P < 0.001). The overall TTR (Day 4-90) was significantly different between the GBWD group and CFD group (56.86 ± 10.72 vs. 52.87 ± 13.92, P = 0.007).In subgroup analysis, the TTR was also significantly different between the GBWD group and CFD group during the first month of treatment (Day 4-14: 54.28 ± 21.90 *vs.* 47.01 ± 26.25, *P* = 0.012; Day 15-28: 59.60 ± 20.12 *vs.* 51.71 ± 18.96, *P* = 0.001). However, no significant difference in the TTR was observed after 29 days of treatment. These data suggest that GBWD provided clinical benefits.

## Introduction

Despite the recent approval of direct oral anticoagulants (DOACs) (Zhang et al., 2014b; Arwood et al., 2016), warfarin remains the most commonly used oral anticoagulant. Warfarin is the only option for patients with artificial heart valves or atrial fibrillation with severe hepatic/renal insufficiency. In particular, warfarin is suitable for people on low incomes or intermediate incomes who cannot afford the high cost of DOACs. However, warfarin administration is hindered by a narrow therapeutic index and large variability among different individuals in the dose required to achieve therapeutic anticoagulation (Arwood et al., 2016).

Studies have suggested that the cytochrome *P450 2C9* (*CYP2C9*) and *vitamin K epoxide reductase complex subunit 1* (*VKORC1*) genotypes are associated with the time needed to achieve therapeutic anticoagulation, the dose requirements of warfarin, and risk of supra-therapeutic anticoagulation and major bleeding (Jonas and Mcleod, 2009; Johnson et al., 2011; Fung et al., 2012; Arwood et al., 2016). Therefore, in 2007, the United States Food and Drug Administration updated the drug label for warfarin to reflect the potential value of incorporating genetic information into dose selection. Patients with certain genetic variants of *CYP2C9* require a lower dose of warfarin and a longer time to reach a stable dose. They are also at higher risk of over-anticoagulation and serious bleeding (Higashi, 2002; Schwarz et al., 2008; Voora et al., 2010). Patients with the A/A haplotype of *VKORC1* have a reduced time to the first International Normalized Ratio (INR) within the therapeutic range and to the first INR > 4 (Higashi, 2002; Schwarz et al., 2008; Voora et al., 2010).

Some randomized controlled trials have evaluated the clinical efficacy of gene-based warfarin administration (Jonas et al., 2013; Kimmel et al., 2013; Pirmohamed et al., 2013; Belley- et al., 2015). However, the results were mixed, with some studies recommending gene-based warfarin therapy and others not supporting this strategy.

We explored the clinical efficacy of gene-based warfarin administration by comparing the anticoagulation control and clinical events related to warfarin with gene-based warfarin dosing (GBWD) to those with clinically fixed dosing (CFD) in a real-world scenario.

## Methods

### Study Design and Eligibility

This was a retrospective cohort study designed to compare GBWD with CFD. The study protocol was approved by the Ethics Committee of China Fujian Medical University Union Hospital (Fujian, China). All patients who were newly prescribed warfarin between March 2014 and May 2019 were enrolled.

The inclusion criteria were people: (i) aged ≥18 years; (ii) with results for detection of *VKORC1* and *CYP2C9* polymorphism available; (iii) in whom anticoagulation with warfarin for ≥3 months had been achieved.

The exclusion criteria were individuals with: (i) a diagnosis of active cancer; (ii) severe infection or respiratory failure; (iii) severe hepatic/renal insufficiency; (iv) hematologic diseases; (v) abnormal thyroid function.

### Data Collection and Follow-Up

The intervention was the initial warfarin dose. GBWD was calculated according to the International Warfarin Pharmacogenetic Consortium (IWPC) algorithm. The CFD of warfarin was 2.5 mg/day. For Chinese patients with mechanical heart valves, bleeding was the major complication rather than thromboembolism. Most clinicians apply low-intensity anticoagulation for patients undergoing heart valve replacement in China (Zhou et al., 2005). Therefore, the target therapeutic INR range was 1.7–2.5 for patients with valve replacement, and 2.0–3.0 for patients with atrial fibrillation or venous thromboembolism (Tao et al., 2018).

Patient interview, review of medical records, and telephone follow-up revealed the following data: age, sex, height, weight, indication for warfarin therapy, range of target INR, date of initiation of warfarin therapy, initial warfarin dose, concomitant medications, INR values, warfarin doses, smoker status, and thromboembolic and bleeding events.

### Genotyping

Peripheral venous blood (2 mL) was collected from each patient. Genomic DNA was extracted using a DNA extraction kit according to manufacturer (Shanghai Baio, Shanghai, China) instructions. The *CYP2C9* and *VKORC1* genotypes were determined by DNA microarray hybridization reactions to a gene chip (Shanghai Baio) after initial polymerase chain reaction amplification of the target region with mutation sites using primers for the major variant alleles CYP2C9*2 (rs1799853), CYP2C9*3 (rs1057910), and *VKORC1 (rs9923231)*. The mutant allele or wild-type allele was identified using a biometric reader (BE 2.0; Shanghai Baio).

### Outcomes

The primary outcomes were the time to achieve the therapeutic INR and time in the therapeutic range (TTR) during a 3-month timeframe. The time to achieve the first INR in the therapeutic range was defined as the time from the initiation of warfarin therapy until the first INR reached the treatment anticoagulation range. TTR was calculated based on the method developed by Rosendaal et al. (1993). The secondary outcomes were INR ≥4 events, major bleeding, minor bleeding, and thromboembolism events (TEs). Major bleeding events are those that result in death, are life-threatening, cause chronic sequelae or consume major health-care resources, as defined in the International Society on Thrombosis and Haemostasis classification (Schulman and Kearon, 2005).

### Sample Size

The primary endpoint of this study is the goal attainment rate of the TTR index. The sample size was calculated according to the expected difference between the TTR goal attainment rate of the clinically fixed dosing group and gene-based dosing group. Based on previous research, the TTRs of the clinically fixed dosing group and gene-based dosing group were about 60.3% and 67.4% (Pirmohamed et al., 2013). If the requirement to meet is at least 80%, a class of errors will be 0.05. Calculation with PASS V.11 software shows that a study of the gene-based dosing group and clinically fixed dosing group at a 1:1 proportion needs at least 268 patients per group. Assuming a dropout rate or loss rate of 20%, each group needs at least 322 patients, with a total of 644 patients.

### Statistical Analyses

Data were analyzed using SPSS v22.0 (IBM, Armonk, NY, USA) and Prism v7.0 (GraphPad, San Diego, CA, USA). Statistical significance was set at *P* < 0.05. Continuous data are given as the mean ± standard deviation. Categorical variables are described as percentages. The chi-square test or Fisher’s exact test (as appropriate) was used to identify the difference between two percentages. A two-tailed Student’s *t*-test was used to compare two sets of continuous data. Time-to-event outcomes were shown with Kaplan–Meier curves.

## Results

### Population Characteristics

Of the 915 patients who were reviewed, 71 were excluded from the analysis: 11 patients had abnormal liver function; 9 did not have an indication for warfarin therapy; five were undergoing chemotherapy; 14 had abnormal thyroid function; 32 switched to other anticoagulant drugs. These exclusions resulted in a final study population of 844 patients. In 413 cases, the initial dose of warfarin was guided by GBWD (IWPC algorithm). In 431 patients, CFD (2.5 mg/day) was employed.

The demographic characteristics of the patients are shown in **Table 1**. Patient characteristics and genotypic distributions were well-balanced between the two groups at baseline. The mean age of the recruited patients was 56.45 ± 11.46 years, and 57.8% of patients were female.

**Table 1:** Demographic characteristics of patients.

Characteristic	Gene-based dosing group (N = 413)	Clinically fixed dosing group (N = 431)	*P*
Age (years)	57.14 ± 11.02	55.80 ± 11.85	0.088
Male	43.1%	41.3%	0.597
Body surface area (m^2^)	1.59 ± 0.17	1.61 ± 0.18	0.106
Current smoker	14.8%	14.4%	0.874
Current use of amiodarone	21.8%	21.1%	0.810
**Indications for treatment**			0.774
Heart-valve replacement	68.8%	71.0%	
Atrial fibrillation	26.4%	24.4%	
Treatment of DVT and/or PE	4.8%	4.6%	
**Concomitant diseases**			
Hypertension	24.0%	23.0%	0.732
Diabetes mellitus	9.9%	9.5%	0.839
***VKORC1* genotype**	86.7%	87.7%	0.657
*AA*	12.1%	11.1%	
*AG*	1.2%	1.2%	
*GG*			
***CYP2C9* genotype**	93.7%	93.5%	0.905
*1/*1	6.3%	6.3%	
*1/*3	0	0.2%	
*3/*3			

DVT, deep venous thrombosis; PE, pulmonary embolism.

The “*” in CYP2C9 genotype means an expression of a gene mutation site.

The indications for warfarin were artificial heart valves, atrial fibrillation, and venous thromboembolism. Also, 93.6% of patients were *CYP2C9* wild-type (CYP2C9*1/*1) and 87.2% were *VKORC1 AA*. The distributions of genotypes were consistent with a Han-Chinese population reported by Zhang and colleagues (Zhang et al., 2016).

### Primary Outcomes

**Figure 1:**
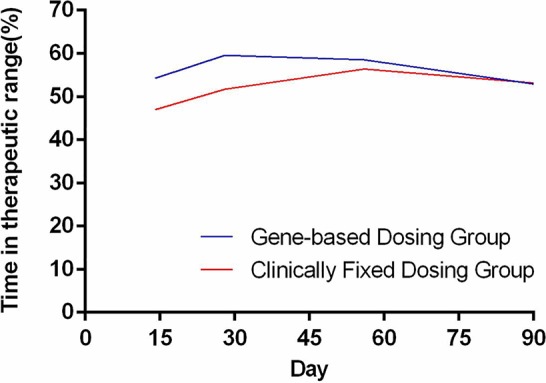
Time needed to reach the therapeutic INR.

**Figure 2:**
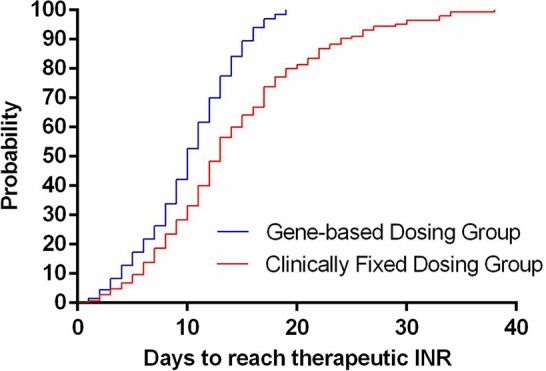
Time in the therapeutic range during follow-up.

The time needed to achieve the therapeutic INR (in days) for patients in the GBWD group was shorter than that for patients in the CFD group (10.21 ± 4.68 *vs.* 14.31 ± 8.26, P < 0.001) (**Table 2**, **Figure 1**). The overall TTR (Day 4–90) was significantly different between the GBWD group and CFD group (56.86 ± 10.72 vs. 52.87 ± 13.92, P = 0.007).In subgroup analysis, the TTR was also significantly different between the GBWD group and CFD group during the first month of treatment (Day 4-14: 54.28 ± 21.90 *vs.* 47.01 ± 26.25, *P* = 0.012; Day 15-28: 59.60 ± 20.12 *vs.* 51.71 ± 18.96, *P* = 0.001). However, no significant difference (P = 0.206, P = 0.887) in the TTR was observed after 29 days of treatment. (**Table 2**, **Figure 2**).

**Table 2:** Primary outcomes (anticoagulation control).

Time	Gene-based dosing group (N = 413)	Clinically fixed dosing group (N = 431)	*P*
Time to reach therapeutic INR -days	10.21 ± 4.68	14.31 ± 8.26	<0.001
Day 4–90	56.86 ± 10.72%	52.87 ± 13.92%	0.007
Day 4–14	54.28 ± 21.90%	47.01 ± 26.25%	0.012
Day 15–28	59.60 ± 20.12%	51.71 ± 18.96%	0.001
Day 29–56	58.55 ± 23.24%	56.42 ± 25.47%	0.206
Day 57–90	52.95 ± 22.52%	53.17 ± 23.07%	0.887

### Secondary Outcomes

Only five major bleeding events and five thromboembolic events were reported, and they occurred in the CFD group. One patient in the GBWD group had minor bleeding events, whereas 34 patients in the CFD group had minor bleeding events. There were significant differences in the prevalence of minor bleeding between the GBWD group and CFD group (0.2% *vs.* 7.9%, *P* < 0.001), but there were no significant differences in INR ≥4.0 events, major bleeding events, or thromboembolic events (*P* > 0.05 for all) (**Table 3**).

**Table 3:** Secondary outcomes.

Time	Gene-based dosing group (N = 413)	Clinically fixed dosing group (N = 431)	*P*
INR ≥4.0	3.4%	5.1%	0.218
Major bleeding events	0	1.2%	0.062
Non-major bleeding events	0.2%	7.9%	< 0.001
Thromboembolic events	0	1.2%	0.062

## Discussion

Since 2013, warfarin-related gene testing has been carried out in increasing numbers of hospitals in China. The IWPC algorithm was introduced in China to recommend the initial dose of warfarin. The IWPC algorithm was based on a study involving the enrollment of >5000 patients from three major ethnic populations (Caucasian, African, and Asian), which was the largest-scale study on warfarin-dose prediction (Wen et al., 2017).

Prompt achievement of therapeutic anticoagulation is a major goal when initiating warfarin treatment (Arwood et al., 2016). Especially for patients undergoing implantation of artificial heart valves, the first postoperative month is a high-risk period for thromboembolism (Baumgartner and Helmut, 2017). The risk of thrombosis recurrence in patients with acute venous thrombosis in the first few months after the diagnosis is also very high (White, 2003; Arwood et al., 2016). The risk of major bleeding events is tenfold higher during the first month following warfarin initiation than for the remainder of therapy (Heit et al., 2003). In China, warfarin is usually started at a fixed dose of 2.5 mg/day, with dose titration based on the INR response (Zhang et al., 2014a). However, achieving the target anticoagulant treatment range is difficult and during this time, patients are at a high risk of thrombosis and bleeding (Pirmohamed et al., 2013; Hua et al., 2018).

We revealed that the use of GBWD improved primary outcomes (the time to achieve the therapeutic INR and TTR) significantly. The time to achieve the therapeutic INR in the GBWD group was shorter than that in the CFD group. The time to reach a therapeutic INR has been studied by several investigators. Our results are in accordance with those of other studies and suggest that a GBWD algorithm may shorten the time to reach a therapeutic INR (Johnson et al., 2017; Wen et al., 2017). However, Li and colleagues, (2013) showed no difference in the time to reach the target INR between a GBWD group and CFD group. The reason for this difference could be related to the loading dose (5 mg/day) used in the CFD group in the study by Li and colleagues.

We showed that the overall TTR (Day 4-90) in the GBWD group was higher than that in the CFD group. Subgroup analysis also revealed that the TTR in the GBWD group was higher than that in the CFD group in the first 28 days. However, the TTR did not differ between the two groups from 29 days to 90 days. These data may suggest that the benefits of GBWD over CFD are especially marked in the first month after anticoagulation initiation. Subsequently, GBWD had less of an effect on anticoagulation control, and multiple-dose titrations might have had a greater role. The finding that the TTR was shorter in the GBWD group is similar to that observed in the GIFT, EU-PACT, and COUMAGEN-II trials (Anderson et al., 2012; Wen et al., 2017) but different from that in the COAG study and other Asian-based studies (Kimmel et al., 2013; Gage et al., 2017; Syn et al., 2018; Zhe et al., 2018). Our study revealed that GBWD resulted in a lower prevalence of bleeding events related to anticoagulation therapy, which may have been due to superior primary outcomes.

All of the primary outcomes and some of the secondary outcomes strongly indicated that GBWD provided benefits. Also, the IWPC algorithm could be suitable for Chinese populations if a locally developed dosing algorithm is not available, a hypothesis that is in accordance with a study by Li and collaborators, (2013). This study suggests that the use of gene-based warfarin dosing deserves continued consideration, evaluation, and application (assuming that the cost of the warfarin plus the genotyping is less than the use of a DOAC) throughout the world.

Our study had three main limitations. First, as a retrospective study, participants were not assigned randomly to the GBWD group and CFD group, so selection bias and other potential confounding variables may have been present. Second, telephone follow-up may not collect all the bleeding and thrombosis-related events. Prospective, multicenter cohort studies are required to confirm our findings. Third, the sample size was too small to perform a subgroup analysis of the effect of CYP2C9 and VKORC1 composite genotypes on warfarin.

## Conclusion

The GBWD group was superior to the CFD group in terms of anticoagulation control and the prevalence of minor bleeding, especially in the first month of initial anticoagulation. These data suggest that GBWD provides clinical benefits. The IWPC algorithm may be suitable for Chinese populations.

## Data Availability Statement

All datasets generated for this study are included in the article/supplementary material.

## Ethics Statement

The study protocol was approved by the Ethics Committee of China Fujian Medical University Union Hospital. The patients/participants provided their written informed consent to participate in this study.

## Author Contributions

JZ analyzed the data and wrote the article, LC designed the research, and TW and WC performed the research. XX and JF collected patient information. All the authors contributed to the final paper.

## Funding

This study was supported by the Natural Science Foundation of Fujian Province, China (grant number 2018Y0037).

## Conflict of Interest

The authors declare that the research was conducted in the absence of any commercial or financial relationships that could be construed as a potential conflict of interest.
